# Microbial response under sulfate stress in a sulfur-based autotrophic denitrification system

**DOI:** 10.3389/fmicb.2025.1615317

**Published:** 2025-06-04

**Authors:** Yiqiang Chen, Xu Jiang, Juanjuan Zhao, Maosheng Yang, Yong Chen, Hong Ling, Yang Liu, Feng Deng, Zhu Wang

**Affiliations:** ^1^Key Laboratory of Environmental Remediation and Ecological Health, Ministry of Industry and Information Technology, Jiangsu Environmental Engineering Technology Co., Ltd, Jiangsu Environmental Protection Group Co., Ltd., Nanjing, China; ^2^Institute of Environmental Research at Greater Bay, Key Laboratory for Water Quality and Conservation of the Pearl River Delta, Ministry of Education, Guangzhou University, Guangzhou, China; ^3^Key Laboratory of Integrated Regulation and Resources Development on Shallow Lakes, Ministry of Education, College of Environment, Hohai University, Nanjing, China; ^4^College of Environmental Science and Engineering, Nanjing Tech University, Nanjing, China

**Keywords:** sulfur-based autotrophic denitrification filter, sulfate salinity, *Thiobacillus*, salinity stress, microbial interactions

## Abstract

This study investigated the responses of the bacterial community structure and metabolic pathways in a sulfur-based autotrophic denitrification filter (SADF) system to fast elevated sulfate salinity, from 0.04 to 1.2% in 30 days. Results showed that the SADF system exhibited robust sulfate salinity stress tolerance at low nitrate concentrations. In the context of sulfate scenarios, the genus *Thiobacillus* significantly proliferated and was identified as the dominant sulfur-oxidizing player in the SADF system, achieving a relative abundance of 63.79% under 1.2% sulfate salinity. Cooperative and competitive interactions were found in the SADF-related microorganisms, promoting stable denitrification performance under high salinity. Surprisingly, with a low hydraulic retention time (HRT) of 60 min, metagenomic sequencing revealed a upregulated abundance of functional genes encoding for enzymes associated with nitrogen and sulfur metabolism, while positive correlations were observed between these two pathways in response to sulfate salinity. Furthermore, global wastewater treatment plants were thoroughly explored for the distribution of the SADF-related microorganisms identified in this study. Interestingly, one-way ANOVA analysis showed that the SADF-related microorganisms were widely distributed globally, demonstrating their universality in potential engineering applications worldwide.

## 1 Introduction

Nitrate, a primary contaminant in wastewater effluents, poses significant ecological and health risks when discharged in excessive quantities (Abascal et al., 2022; Xu et al., 2025). Overloading of nitrate into aquatic systems can trigger severe eutrophication, characterized by algal blooms and hypoxic conditions, ultimately disrupting aquatic ecosystems (Wang L. et al., 2023; Wang T. et al., 2023; Yue et al., 2023). Beyond environmental concerns, elevated nitrate levels in drinking water sources directly threaten human health, contributing to potential health issues such as methemoglobinemia and increased risk of certain cancers (Picetti et al., 2022; Liu et al., 2024). The effective removal of nitrate from wastewater is therefore critical to mitigate environmental degradation and safeguard public health.

Biological denitrification, a microbial mediated process that functions under oxygen-limited conditions (DO <0.5 mg/L), could achieve complete reduction of nitrate to nitrogen gas (N₂) through two distinct metabolic pathways: (1) heterotrophic denitrification (Shao et al., 2025) utilizing organic carbon sources (e.g., methanol, acetate) and (2) autotrophic denitrification (Ye et al., 2024) driven by inorganic electron donors (e.g., H₂, SO). While this process provides an effective nitrogen removal strategy for wastewater treatment, conventional heterotrophic denitrification poses significant limitations, including (1) elevated operational costs from continuous organic carbon supplementation (Xi et al., 2022), (2) excessive sludge production (Wang et al., 2021), and (3) risks of secondary pollution due to residual chemical oxygen demand (COD) in effluents (Sun et al., 2017).

In alignment with global carbon neutrality goals, autotrophic denitrification has emerged as a promising alternative due to its low operational costs, reduced sludge yield, and minimal organic carbon residues. Among autotrophic denitrification technologies, SADF system has attracted increasing attention due to its pronounced advantages over conventional heterotrophic denitrification, including a 55% reduction in sludge generation, over 87% reduction in treatment costs, and significant decreases in greenhouse gas emissions (e.g., carbon dioxide and nitrous oxide) by 80–100% (Zheng et al., 2024; Wang L. et al., 2023; Wang T. et al., 2023; Pang and Wang, 2021).

Recent studies have demonstrated the feasibility of the sulfur autotrophic denitrification (SADN) process for treating nitrate-laden wastewater, achieving satisfactory nitrogen removal performance with total nitrogen removal efficiencies consistently exceeding 90%. For example, Zhao et al. (2024) invented a special filler based on combination with sulfur and siderite, achieving robust removal rates of 91.2% at an extreme cold temperature of 7°C. Li R. et al. (2021) and Li W. et al. (2021) successfully removed both arsenite and nitrate in the SADF system, with the maximum removal efficiency of nitrate at 98.42%. Bai et al. (2023) fused FeS and S with 2:1 as a filler, finding that the optimal hydraulic retention time (HRT) to be 3 h with a removal rate of 90–100%. These findings underscore the effectiveness of SADN as an eco-friendly technology for achieving sustainable wastewater treatment, particularly in scenarios with extreme low C/N ratio and expectations of low carbon emission.

Industrial wastewater from petroleum refining, pharmaceutical manufacturing, and textile dyeing processes typically contains high salinity, primarily composed of sodium chloride (NaCl) and sodium sulfate (Na₂SO₄). The performance of the SADN process varies significantly under different NaCl salinities when treating saline wastewater. Notably, the performance of the SADN process could be improved within a NaCl salinity range from 0.1 to 1.5%. For instance, previous study (Shen et al., 2023) reported a significant improvement in nitrate removal efficiency from 81 to 88%, while the NaCl salinity increased from 0.16 to 1.5%. These findings (Wang T. et al., 2024; Wang X. et al., 2024; Li Y. Y. et al., 2023; Li Z. R. et al., 2023; Chen B. et al., 2022; Chen H. et al., 2022) highlights the adaptability of the SADN process to moderate NaCl salinity levels, making it a promising approach for treating saline industrial wastewater. However, while chloride salts (e.g., NaCl) primarily exert osmotic stress on microbial cells, sulfate salts (e.g., Na₂SO₄) not only impose similar osmotic pressure but may also cause more complex and severe inhibitory effects due to the toxicity of their metabolic by-products (e.g., H₂S, SO₄^2−^) (Chatla et al., 2023). These differences suggest that sulfate-induced salinity may interfere with microbial communities through stronger electron acceptor competition, metabolic pathway disruption, and dramatic shifts in microbial structure.

Previous studies have primarily focused on the effects of NaCl salinity on SADN processes in wastewater treatment systems. Sulfate generated as a byproduct of SADN processes can significantly disrupt the chemical equilibrium under excessive conditions. Therefore, investigating the impact of sulfate-mediated salinity on the SADN process is of great importance, especially for optimizing nitrogen removal performance in high-sulfate industrial wastewater. However, there is limited understanding of the responses of the bacterial community structure and metabolic pathways of SADN process when applied to treat industrial wastewater characterized by high sulfate salinity.

In this study, a SADF system packed with elemental sulfur (S0) as the sole electron donor was specifically designed to thoroughly assess the effects of various Na₂SO₄ salinities (0.04–1.2%) on the performance of the SADN process and its underlying metabolic mechanisms. To our knowledge, no prior studies have specifically examined the effects of Na₂SO₄ salinity, unlike NaCl salinity, on the SADF system, especially with a fast elevated salinity in 30 days and a low HRT of 60 min. The key research aspects include: (1) assessing the robust tolerance to high salt concentrations and resistance mechanisms within the SADF system, (2) investigating the shift in microbial community structure with increasing Na₂SO₄ salinity, (3) analyzing the competitive and cooperative interactions among different microorganisms in the SADF system, (4) examining the upregulated fluctuations in the abundance of functional genes involved in nitrogen and sulfur metabolism under Na₂SO₄ salinity stress, (5) evaluating the global distribution of SADN-related microbial communities across wastewater treatment plants. This comprehensive investigation aims to provide a deeper understanding of the interplay among Na₂SO₄ salinity, microbial dynamics, and metabolic response in SADF systems.

## 2 Materials and methods

### 2.1 Experimental setup and bioreactor operation

A lab-scale upflow packed-bed SADF bioreactor was constructed with a working volume of 2 L (Supplementary Figure 1). The bioreactor was filled with spherical filler, which was prepared by mixing elemental sulfur and calcium carbonate into a uniform granule with a particle size of 3–5 mm at a ratio of sulfur to alkali source of 3:1 (wt./wt.). The reactor was maintained at a constant temperature of 25°C via a water bath, and the hydraulic retention time (HRT) was controlled at 60 min using a peristaltic pump to ensure consistent flow rates throughout the experiment. The seed sludge used was sourced from the anoxic process of a municipal wastewater treatment plant in Jiangsu Province. Initially, the sludge was subjected to intermittent feeding, followed by a gradual transition to continuous feeding over a period of 2 weeks. During this time, the concentration and organic loading rate progressively increased to acclimate the microbial community. Prior to the formal experimental phase, the system had stably operated for 3 months, ensuring that the seed sludge was well-adapted.

To investigate the effects of Na₂SO₄ salinity stress on denitrification performance, the reactor was operated continuously for 30 days, comprising five stages (S0, S1, S2, S3, and S4), each lasting 6 days. During these stages, the salinity (expressed as total dissolved solids, TDS) was set at 400, 3,000, 6,000, 9,000, and 12,000 mg/L, respectively. Reagent-grade Na₂SO₄ was added to the influent to adjust the salinity levels.

The influent NO_3_^−^-N concentration was set to be 20 mg/L. Each liter of synthetic wastewater contained the following components: 145 mg of KNO_3_, 151.2 mg of NaHCO_3_, 3.821 mg of NH_4_Cl, 6.6 mg of K_2_HPO_4_ and 12 mg/L of MgCl_2_. The influent pH was tightly regulated within the range of 7.9 to 8.4 through the combined buffering action of calcium carbonate packing and external supplementation with sodium bicarbonate (NaHCO_3_).

### 2.2 Chemical analysis

Water samples were routinely collected from the SADF system for chemical analysis. Subsequently, they were filtered through 0.45 μm membrane filters. A water quality analyzer (LH-3BAV12, Lianhua, China) was employed to determine the total nitrogen, nitrate nitrogen, and nitrite nitrogen. A multi - function portable meter (DZS-708 L, Leici, China) was used to directly measure the pH, dissolved oxygen, conductivity, and total dissolved solids (TDS).

Regarding the description of the filler’s surface morphology, samples were taken from S0 to S4 and fixed with 2.5% glutaraldehyde (pH = 6.8) at 4°C for 1.5 h. After that, the filler samples were rinsed three times with 50 mmol·L^−1^ phosphate buffer solutions. Then, a series of ethanol washes were conducted at concentrations of 50, 70, 80, and 90%, each wash lasting 15 min. Subsequently, three additional 15-min washes with 100% ethanol were carried out. Next, dehydration was performed using a 1:1 mixture of ethanol and isoamyl acetate for 15 min. Finally, the samples were dehydrated with isoamyl acetate for 15 min. After freeze-drying and gold sputtering, the treated samples were attached to a scanning electron microscope (SEM, FEI Quanta FEG250, USA) for observation.

### 2.3 Metagenomic sequencing and data analysis

Sludge samples were, respectively, collected from stages S0 to S4 with salinities of 400, 3,000, 6,000, 9,000, and 12,000 mg/L. These samples were immediately frozen using dry ice and then shipped to Novogene (Tianjin, China) for library construction and subsequent metagenomic sequencing. DNA sequencing was carried out using the Illumina Novaseq 6,000 PE150 platform. Metagenomic sequencing for each sample yielded 10 Gb raw data. Clean reads from all samples were pooled, co-assembled and binned using metaWrap v1.3 (Uritskiy et al., 2018), which ultimately resulted in 68 bins. Taxonomy annotation was performed using GTDB-tk v2.2.3 software (Chaumeil et al., 2020), and the relative abundance calculation was calculated with coverm v0.6.1 software.^1^ The assembled contigs were aligned with the KO (KEGG Orthology) database (Kanehisa et al., 2016) to annotate the functional genes.

Taxonomy abundance plots and heatmaps were created using R v4.3.0^2^ in conjunction with the ggplot2 package. The nitrogen and sulfur metabolic pathways were depicted based on the KEGG pathway. A co-occurrence network was constructed using R v4.3.0 to illustrate the interaction relationships among the major microbial components in the SADF system, and data visualization was accomplished using Gephi v.0.10.1 software.

### 2.4 Global mapping for SADF-related microorganisms

High-throughput DNA sequencing data from 472 global activated sludge samples were downloaded from the National Center for Biotechnology Information (NCBI) database.^3^ These samples were sourced from 57 wastewater treatment plants (WWTPs) across 39 cities in 12 countries spanning 4 continents. The assembled bins in this study were aligned with the 472 downloaded global samples using the coverm v0.6.1 software. To analyze the geographic differences, the mapped percentages among the 57 WWTPs were calculated, and a one-way ANOVA test was conducted using SPSS software (v26.0).

## 3 Results and discussion

### 3.1 SADF system exhibited robust Na₂SO₄ salinity tolerance

In conventional saline wastewater treatment systems, influent undergoes preliminary desalination via physicochemical pretreatment (e.g., coagulation-flocculation or evaporation) to maintain salinity below the 1.5% (15,000 mg/L TDS) operational threshold for biological processes (Yadai and Suzuki, 2023; Kavitha et al., 2019). To systematically evaluate Na₂SO₄ salinity impacts, we therefore designed a controlled gradient series spanning 400 mg/L (baseline), 3,000 mg/L, 6,000 mg/L, 9,000 mg/L, and 12,000 mg/L, strategically aligned with typical industrial discharge concentrations to bridge laboratory findings and field applications.

**Figure 1:**
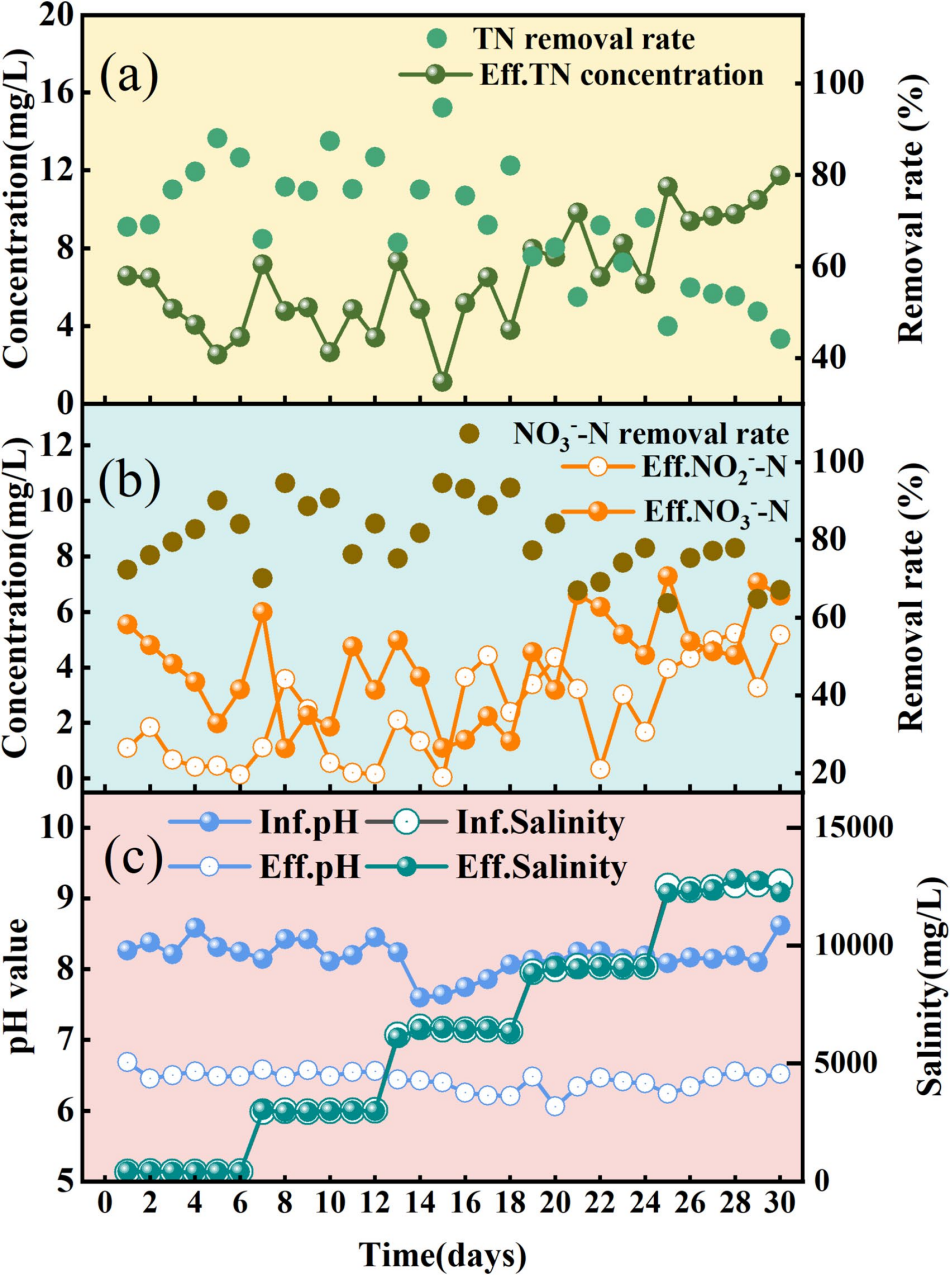
SADF system performance under sulfate salinity stress. **(a)** Effluent TN concentrations and TN removal rate. **(b)** Effluent NO_3_^−^-N, NO_2_^−^-N and NO_3_^−^-N removal rate. **(c)** Influent and effluent pH and salinity.

As illustrated in Figure 1a, during the initial acclimation phase (S0, Days 1–6), the SADF system achieved rapid start-up under low-salinity conditions (TDS = 400 mg/L), attaining an effluent total nitrogen (TN) concentration as low as 3.4 mg/L by the end of S0. Subsequent incremental salinity increases to 6,000 mg/L in phases S1 and S2 exhibited minimal impact on nitrate removal performance, maintaining average effluent NO₃^−^-N concentrations of 3.2 mg/L (S1) and 2.4 mg/L (S2). Notably, nitrite accumulation showed progressive elevation from 0.8 mg/L (S0) to 2.3 mg/L (S2), as depicted in Figure 1b. At the maximum salinity condition (S4: TDS = 12,000 mg/L), the system experienced a marked performance decline, characterized by a 35% reduction in total nitrogen (TN) removal efficiency (from 77% at S2 to 50% at S4) (Figure 1a) and a concurrent 17% decrease in nitrate removal efficiency (from 88 to 71%) (Figure 1b). Moreover, nitrite accumulation demonstrated a progressive increase, culminating in an average concentration of 4.5 mg/L (Figure 1b). The sustained accumulation of nitrite may exert inhibitory effects on microbial metabolism, particularly under high salinity conditions, where intensified redox stress could impair the activity of key metabolic enzymes and disrupt the denitrification pathway, ultimately compromising process efficiency (Bian et al., 2024). Moreover, as an intermediate metabolite, elevated nitrite levels may drive shifts in microbial community composition, rendering the system more susceptible to environmental perturbations (Wang T. et al., 2024; Wang X. et al., 2024).

This observation aligns with findings from previous studies on sulfate-induced inhibition of sulfur-based autotrophic denitrifying bacteria. A recent study (Zhu et al., 2023) demonstrated that denitrification was suppressed and nitrite accumulation occurred when sulfate concentrations exceeded 6,000 mg/L. Notably, another study (Campos et al., 2008) reported an earlier inhibition threshold, observing that denitrification activity began to decline at 1,500 mg/L SO₄^2−^ and was reduced by 85% at 15,000 mg/L SO₄^2−^. Interestingly, Chung et al. (2014) observed no apparent inhibitory effect on autotrophic denitrification even when sulfate concentrations were over 10,000 mg/L, with complete nitrate removal still achieved under these conditions. The observed discrepancies may arise from variations in experimental design, including the use of elemental sulfur (S0) as the electron donor in our study compared to thiosulfate (S₂O₃^2−^) in other’s systems and our continuous-flow operation with a HRT of only 60 min, contrasting with their batch-mode operation utilizing flask bottles with a prolonged incubation period of up to 350 h.

Throughout the experimental process, the influent pH remained consistently at an average of 8.18, while the effluent pH remained relatively stable at an average of 6.44 (as shown in Figure 1c). This stability in effluent pH was achieved by supplementing the system with sodium bicarbonate (NaHCO₃) to neutralize the acidity generated during sulfur oxidation.

In general, the SADF system demonstrated exceptional operational resilience under two challenging conditions: (1) rapid Na₂SO₄ salinity escalation (400 to 12,000 mg/L TDS within 30 days) and (2) a comparatively low HRT of 60 min. Despite these stressors, the system maintained a 71–88% nitrate removal efficiency alongside sustained high volumetric nitrate loading rate (0.26–0.32 kg NO₃^−^-N/m^3^·d). Given that HRT and prolonged salt-tolerant sludge acclimation are critical operational parameters for bioreactor performance in hypersaline wastewater treatment, we hypothesize that optimizing HRT and gradual microbial adaptation could further enhance TN removal in scenarios involving nitrate-laden wastewater with elevated Na₂SO₄ salinity. These findings not only highlight the significant potential of this approach but also provide a theoretical foundation for optimizing treatment strategies in saline wastewater management, thereby warranting comprehensive investigation through future experimental validation studies. However, despite the good denitrification performance and tolerance of the SADF system under high sulfate concentrations, the elevated sulfate levels may pose potential impacts on downstream treatment units. These impacts include changes in microbial community structure and corresponding alterations in metabolic pathways. Additionally, high sulfate concentrations can accelerate the dissolution of calcium carbonate fillers, damage their structural stability, and weaken their buffering capacity. Therefore, while the SADF system shows promise for treating high-sulfate wastewater, these potential downstream effects should be carefully considered in the design and operation of integrated treatment processes.

### 3.2 The fluctuation in denitrification performance was attributed to disturbance in the microbial community structure

Through SEM analysis, microbial colonization and biofilm formation on the surface of the filler were observed under different salinity stress conditions. At 500x magnification, the surface of the new, unused filler was uneven, featuring numerous grooves and irregularities (Supplementary Figure 2). After start-up at a salinity of 400 mg/L, the filler surface exhibited depressed cavities and numerous irregular cracks. This phenomenon indicated that after reacting with nitrate, the filler provided a larger specific surface area for biofilm growth, enhancing denitrification and significantly accelerating the biofilm formation rate in wastewater treatment (Zhou et al., 2021).

Upon further magnification of the S0 filler surface to 10,000x, numerous short rod-shaped, long rod-shaped, and spherical bacteria were observed on the filler surface, suggesting a gradually stabilizing biofilm structure. Additionally, some filamentous bacteria were detected on the filler biofilm. These filamentous bacteria could act as a bridge connecting various bacteria, facilitating the colonization of diverse microorganisms on the membrane surface (Secor et al., 2015). With fast elevated salinity, the microbial density on the filler surface also rose. Interestingly, SEM observations revealed that the microbial composition on the surface was similar across different salinities, mainly consisting of rod-shaped and spherical bacteria. However, the number of filamentous bacteria gradually decreased, indicating that high salinity had a certain inhibitory effect on the growth of filamentous bacteria (Quartaroli et al., 2019). For the S3 and S4 filler samples, the biofilm on the surface became much denser, with a further increase in the number of short rod-shaped and spherical bacteria. These results suggested that the sulfur-based filler supported a relatively rich microbial population after continuous operation. Nevertheless, the microbial structure seemed to change significantly with increasing salinity. Meanwhile, a more detailed investigation of the taxonomic changes in the main functional microorganisms and the microbial community structure at each stage of the reactor was required. Subsequently, metagenomic sequencing was employed for downstream microbial analysis at the genomic and genetic levels.

**Figure 2:**
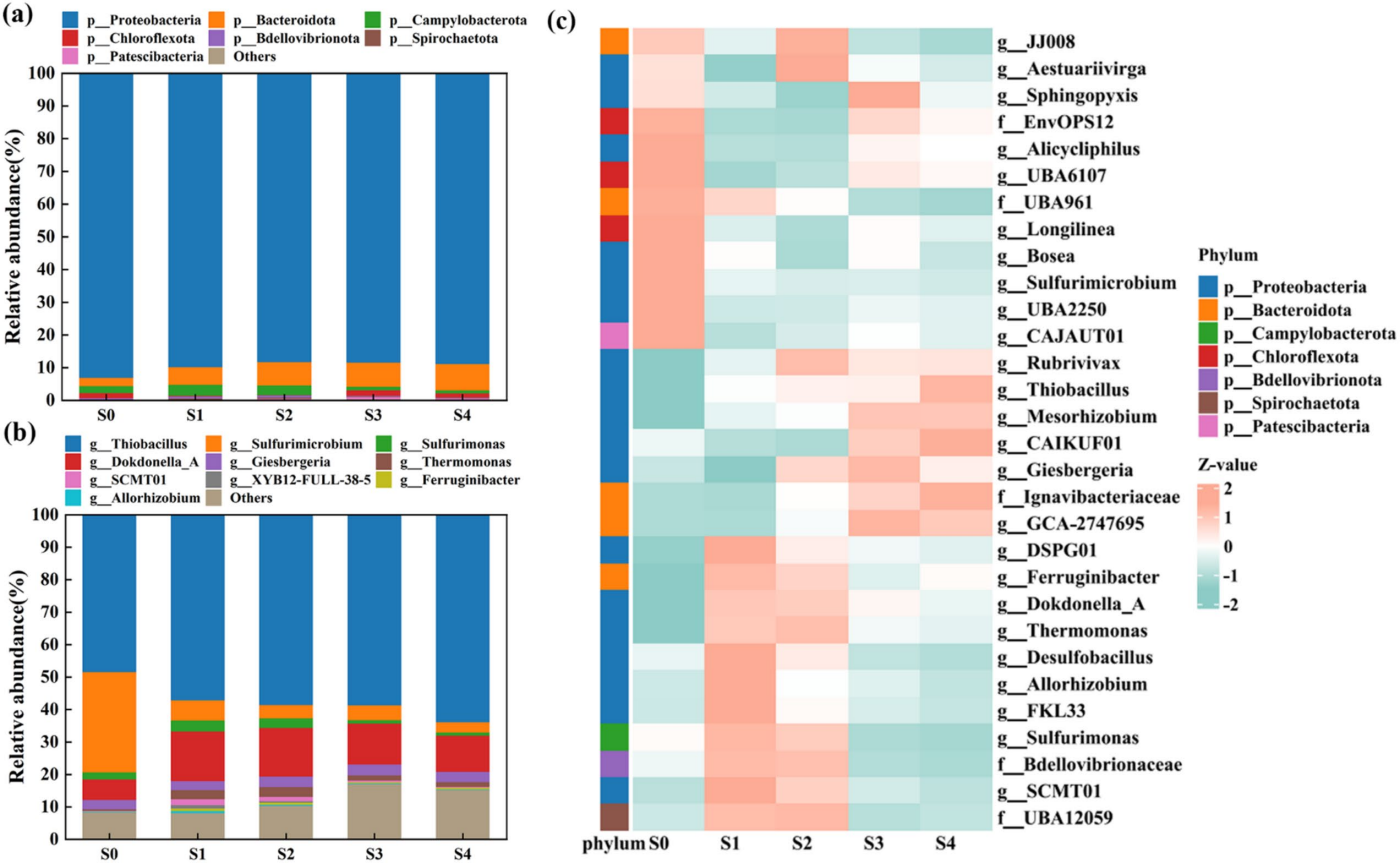
Microbial community shift at five salinity stages at the **(a)** phylum level and the **(b)** genus level. **(c)** Heatmaps of the top 30 abundant bins with standardized Z score corresponding to the relative abundance.

At the phylum level (Figure 2a), Proteobacteria was the most abundant phylum, accounting for 88.2–93.0% of the entire microbial community. This was not unexpected, as many denitrification microorganisms belong to Proteobacteria (Yang et al., 2021). Bacteroidota, the second most abundant phylum, was also found to be salinity-resistant, with its relative abundance increasing from 2.5 to 8.1%. These two phyla have been previously identified as typical phyla involved in autotrophic denitrification processes and were also shown to exhibit selectivity to NaCl salinity (Li Y. Y. et al., 2023; Li Z. R. et al., 2023).

At the genus level (Figure 2b), it was obvious that salinity stress remarkably shaped microbial community structure. At stage S0, the top three dominant genera were *Thiobacillus* (48.37%), *Sulfurimicrobium* (30.90%), and *Dokdonella_A* (6.30%), all of which were denitrifiers. Interestingly, as salinity increased, the relative abundances of SADF-related bacteria showed opposite trends. For example, in our SADF system, there were three main genera that utilized elemental sulfur as an electron donor for sulfur autotrophic denitrification: *Thiobacillus*, *Sulfurimicrobium*, and *Sulfurimonas*. The abundance of *Thiobacillus* increased from 48.37% (at S0) to 63.79% (at S4), indicating that this genus was highly resistant to salinity stress and played a key role in nitrogen removal in the SADF system. This might be attributed to its salt-tolerance genes (Li R. et al., 2021; Li W. et al., 2021). Notably, regardless of the salinity stress, the dominant genus *Thiobacillus* maintained the highest abundance, and its proportion was positively correlated with salinity (*R*^2^ = 0.84, Supplementary Figure 3). Similar results were also observed in the previous study (Xia Y. et al., 2019; Xia Z. et al., 2019), where both studies noted the changes in denitrifying bacteria under different salinity conditions. Under high salinity conditions, the relative abundance of certain denitrifying bacteria increased, showing strong salt tolerance.

In contrast, the relative abundances of *Sulfurimicrobium* and *Sulfurimonas* decreased from 30.90 to 3.23% and from 3.35 to 0.95%, respectively. It could be inferred that these two genera might be more sensitive to salinity stress. Overall, the total abundances of these three genera reached 81.43% at a salinity of 400 mg/L and then rapidly dropped to 66.65% at a salinity of 3,000 mg/L. Surprisingly, their abundances remained relatively stable in the later stages, even showing a slight increase to 67.97% at a salinity of 12,000 mg/L. A high proportion of sulfur-oxidizing bacteria might significantly contribute to the fluctuating yet relatively high nitrogen removal efficiency observed in the SADF system. Additionally, Figures 2a,b indicated that the top 10 most abundant bacteria in the system were all rod-shaped bacteria, while cocci or filamentous bacteria were not commonly detected in any of the five stages. This was slightly different from the SEM results, suggesting that the spherical morphology observed in the SEM images might not be cocci but could be extracellular polymeric substances (EPS) (Zhang et al., 2021).

As shown in Figure 2c, it was clear that the standardized Z score of almost all the top 30 abundant bins underwent significant changes under the influence of salinity. Moreover, a distinct pattern change was observed at stage S3, where salinity-resistant bacteria began to predominate, indicating that the increase in salinity to 9,000 mg/L had a crucial impact on the system (Chen B. et al., 2022; Chen H. et al., 2022). In this study, under low nitrogen loads, the shift in the microbial community structure in response to salinity stress influenced the denitrification performance in the SADF system.

### 3.3 Competition and cooperation among different microorganisms enhance salinity resilience in the SADF system

To gain deeper insights into the symbiotic relationships among SADF-related microorganisms, co-occurrence network was specifically constructed for 4 abundant autotrophic and heterotrophic denitrifiers, namely *Thiobacillus*, *Sulfurimicrobium*, *Thermomonas* and *Dokdonella_A*, to illustrate their interactions with other bins. Bins connected to these genera were mainly classified into six phyla, among which Proteobacteria and Bacteroidota were most widely distributed, accounting for over 77% of all nodes.

**Figure 3:**
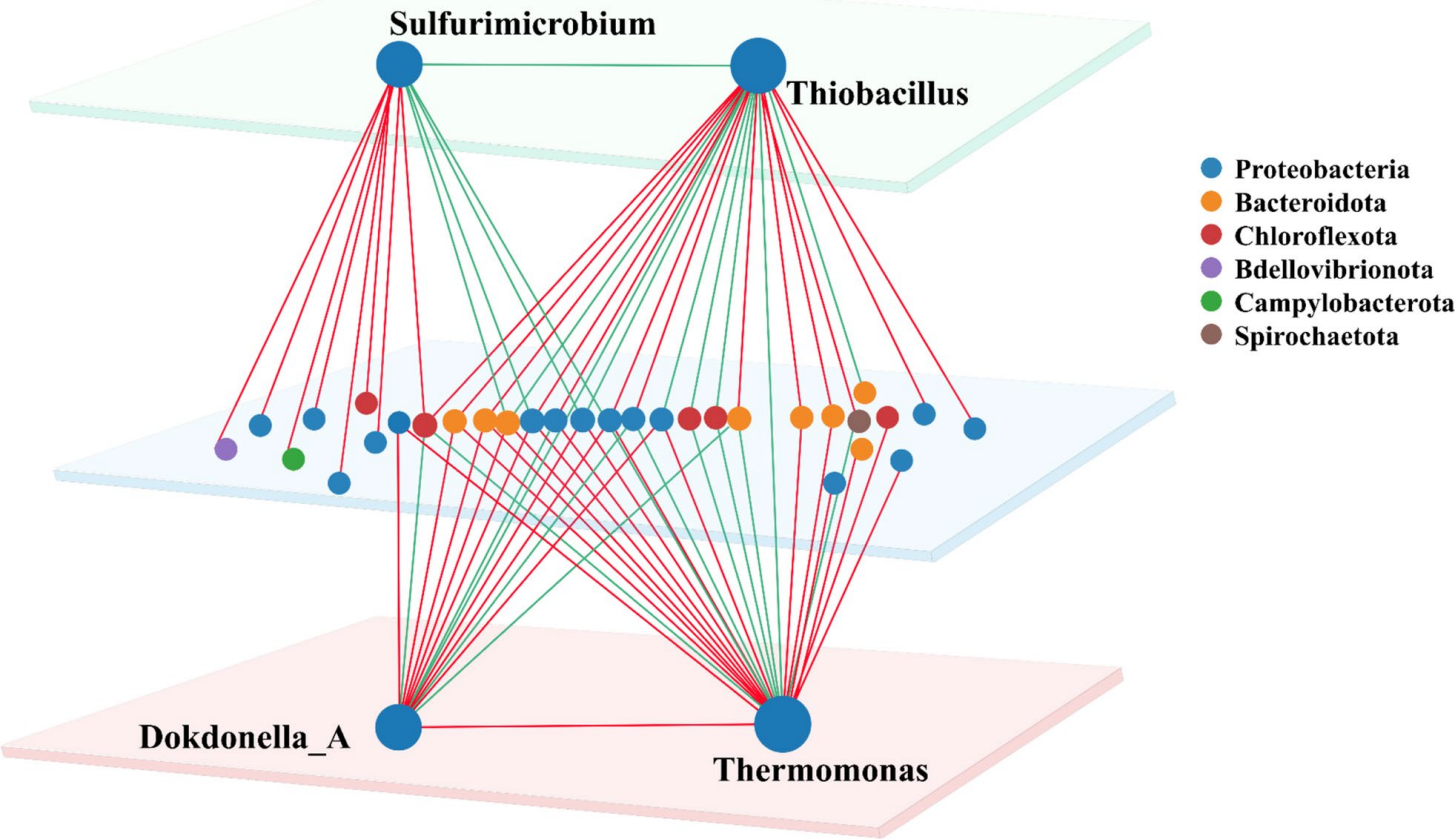
Co-occurrence network for 4 major autotrophic and heterotrophic denitrifiers. (Node size represents the abundance of symbiotic bins. Red colour indicates the positive correlation, while the green color for the negative correlation).

As the most abundant denitrifier, *Thiobacillus* was found to have 13 positive and 9 negative correlations with other bins (Figure 3). Notably, *Thiobacillus* exhibited complex relationships with other denitrifiers. For instance, it was positively correlated with *Ferruginibacter* and unclassified genera belonging to *Ignavibacteriaceae*, which could be explained by the salinity resistance of these bins (Figure 2c). Previously, *Ferruginibacter* was reported to be related to biofilm formation (Xia Y. et al., 2019; Xia Z. et al., 2019) and *Ignavibacteriaceae* was found to be capable for dissimilatory nitrate reduction to ammonia (DNRA) through nitrite (Lawson et al., 2017). Surprisingly, the other 3 denitrifiers, *Dokdonella_A*, *Sulfurimicrobium* and *Thermomonas* were all negatively correlated with *Thiobacillus*, possibly due to the competition for the same substrate and ecological niche (Pastore et al., 2021).

*Sulfurimicrobium* was the second most abundant bacterium at S0, but its abundance rapidly declined under sulfate salinity stress, which was previously identified as an autotroph utilizing thiosulfate, tetrathionate, and elemental sulfur as an electron donor (Kojima et al., 2021). In the co-occurrence network, *Sulfurimicrobium* was positively correlated with another sulfur-oxidizing denitrifier *Sulfurimonas*, whose abundance also decreased significantly as salinity increased. This is consistent with the results reported in studies on salinity stress that salinity inhibits sulfur metabolic activity, indicating that these microbial communities generally face the problem of limited electron donor utilization in high-salinity environments (Zhou et al., 2024). *Thermomonas* and *Dokdonella_A* were likely to be involved in heterotrophic denitrification in WWTPs. However, *Thermomonas* was also identified to possess an autotrophic pathway in the mixotrophic denitrification system (Xing et al., 2018). These two heterotrophic genera had a positive relationship in our SADF system, which was consistent with the previous study that *Thermomonas* and *Dokdonella_A* were, respectively, responsible for nitrate and nitrite reduction (Wang et al., 2019).

The microbial co-occurrence network for the 4 major denitrifiers in the SADF system reveals intricate patterns of competition and cooperation, which may contribute to the system’s enhanced denitrification stability under salinity stress. The predominance of positive over negative correlations suggests that cooperative interactions among microorganisms may outweigh competitive ones within the SADF system. This tendency may reflect a collective microbial strategy to cope with salinity stress, in accordance with interaction patterns observed in response to temperature fluctuations (Zhang et al., 2022). Such correlations may indicate similar ecological responses to environmental changes. However, these inferences remain speculative and require further validation through experimentally verified gene expression and metabolic dynamics.

### 3.4 Stimulation of nitrogen and sulfur metabolic pathways under sulfate stress

**Figure 4:**
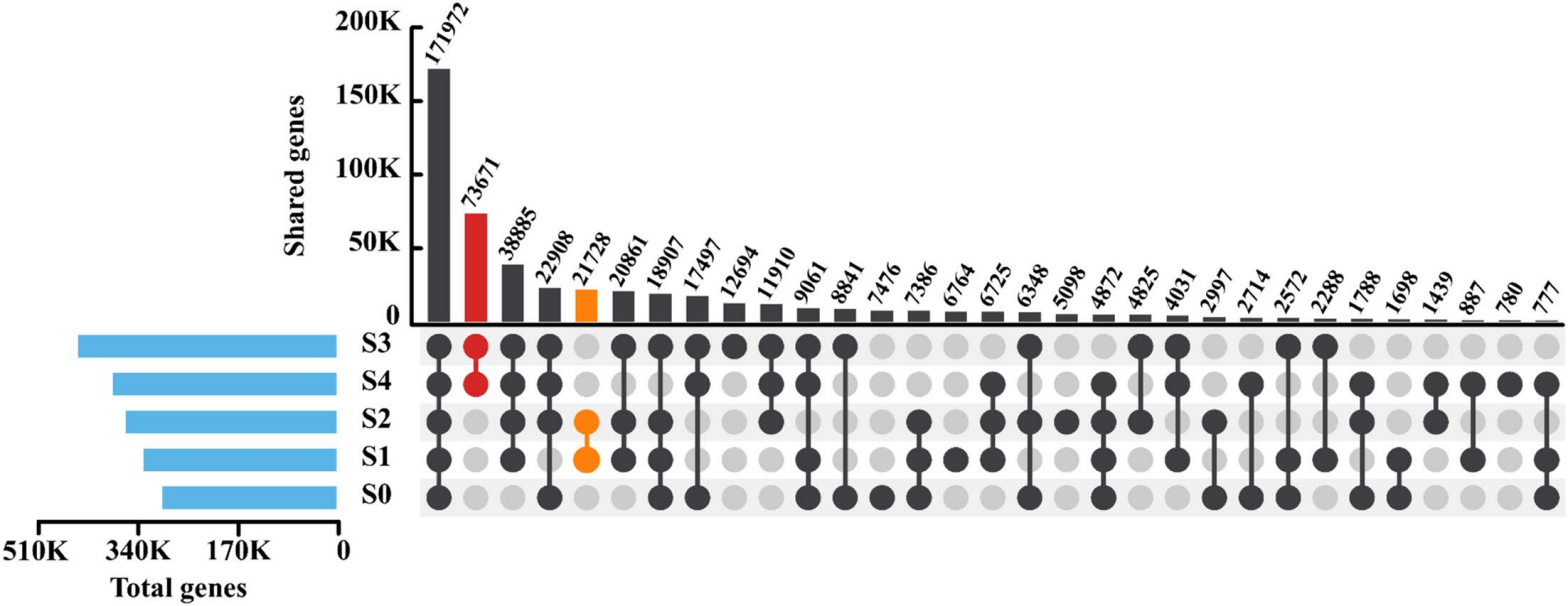
Microbial similarity based on gene patterns at 5 different salinity stages.

To explore the microbial similarities among samples with varying salinities, the distribution of gene counts and the similarity in correlation analysis based on genetic composition were investigated accordingly. As depicted in Figure 4, the number of genes increased with rising salinity, reaching a peak at a salinity of 9,000 mg/L. Nevertheless, when the salinity reached 12,000 mg/L, the gene count decreased, suggesting that extremely high salinity may impede gene expression in microorganisms. Surprisingly, a total of 171,972 genes were shared among all samples, accounting for 59.75, 53.84, 49.33, 40.25, and 46.49% of the total genes in each respective sample. This indicated that despite significant variations in salinity, the SADF system largely shared similar functional genes. Regarding unique genes, the five samples had 7,476, 6,764, 5,098, 12,694, and 780 unique genes, respectively. It was observed that the number of unique genes in S4 was significantly lower than that in samples with other salinities, suggesting that high salinity might suppress gene expression. The varying percentages of unique genes could be attributed to shift in the microbial community composition, which may potentially cause fluctuations in the denitrification performance of the SADF system (Wang et al., 2018).

In pairwise comparisons, it was notable that S3 and S4 shared a relatively large number of 73,671 genes, while S1 and S2 shared 21,728 genes. Both values were significantly higher than the number of shared genes in other pairs. This result was consistent with the findings in Supplementary Figure 4, where S1 and S2, as well as S3 and S4, exhibited strong correlations. Additionally, the correlation between S0 and other samples was relatively weak, indicating that sulfate salinity had a significant impact on the genetic composition in the SADF system.

**Figure 5:**
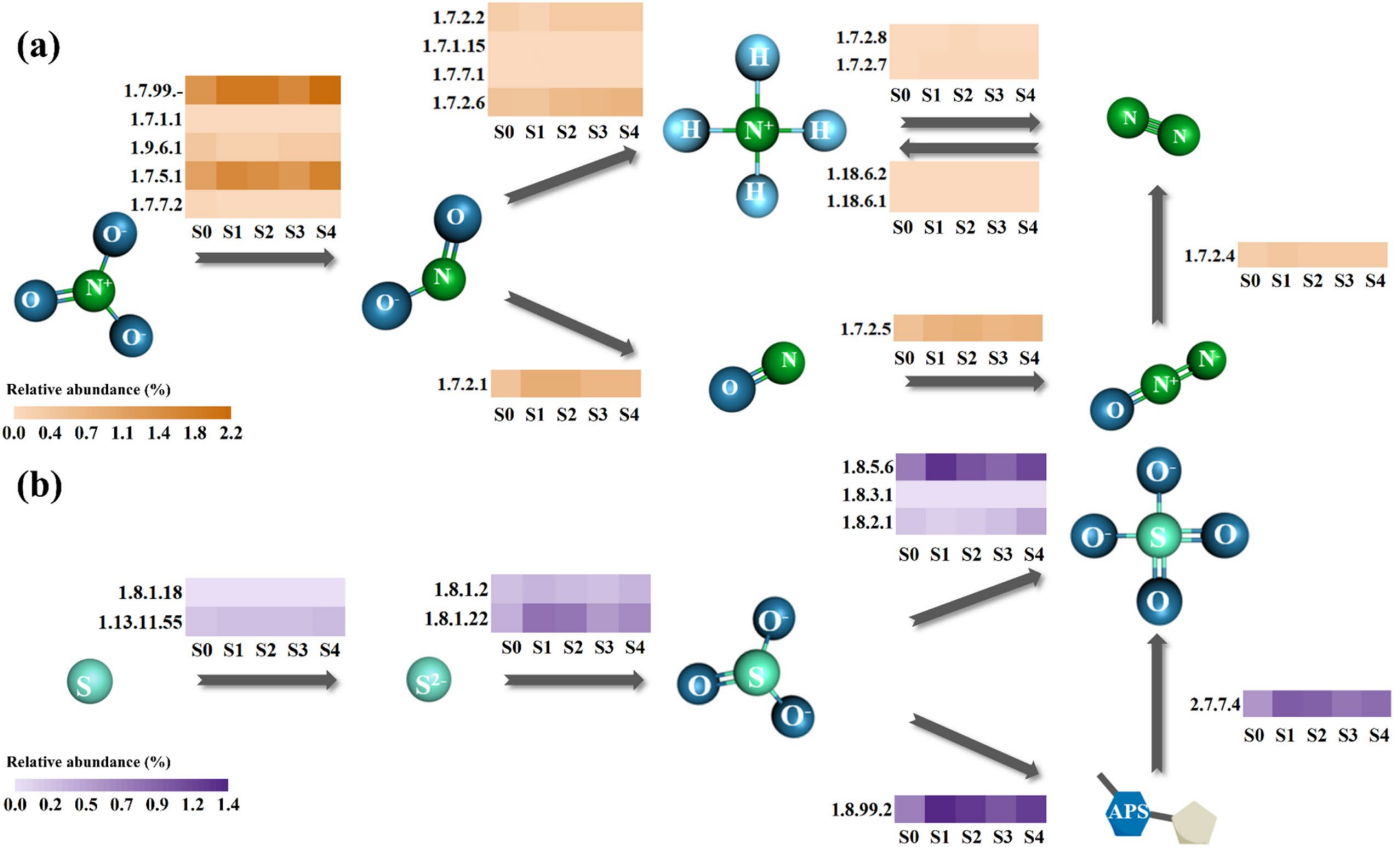
Nitrogen **(a)** and sulfur **(b)** metabolic pathways of the SADF system. The scale bar represents the relative abundance. Numbers near the box represent EC numbers for each enzyme.

As mentioned above, the number of genes escalated with the salinity increase. However, it remains to be resolved whether the abundances of related enzymes in the nitrogen and sulfur metabolic pathways changed in the same direction. Based on the KEGG database, the nitrogen and sulfur metabolic pathways in the SADF system were reconstructed. Among all the genes shown in Figure 5, genes encoding nitrate reductase 1 (EC 1.7.99.-) exhibited the highest relative abundance (1.35–2.16%) in both the nitrogen and sulfur metabolic pathways.

Regarding the nitrogen metabolic pathway, 12 out of 16 N-related genes identified in S0 showed varying degrees of increase under high salinity stress. The total N-related gene abundance increased from 4.92% in S0 to 7.28% in S4. Interestingly, among various nitrogen metabolic steps, the relative abundance of nitrate reductase genes increased most notably (from 2.94 to 4.25%), suggesting that the conversion of nitrate to nitrite may be particularly sensitive to rapidly increasing salinity (Jeong et al., 2018).

Simultaneously, 7 out of 9 genes related to sulfur metabolic pathway showed an increasing trend in relative abundance in response to salinity stress, with the total abundance rising from 3.03% at S0 to 4.87% at S4. There were mainly two pathways for the transformation of elemental sulfur to sulfate, one generating APS while the other directly produced sulfate from sulfite (Wu et al., 2021). Results showed that at different salinity levels, the total enzyme abundance for the former pathway (1.24–2.38%) was consistently higher than the latter one (0.97–1.55%), suggesting that APS was an important intermediate in the SADF system. Surprisingly, despite the high external sulfate concentrations, the relative abundance of genes involved in the sulfite-to-sulfate conversion pathway also increased, suggesting a potential metabolic adaptation under hypersaline conditions.

Overall, the total relative abundance of genes related to nitrogen and sulfur metabolic pathways exhibited similar upregulated patterns with the fast elevated salinity, with positive correlations identified (*R*^2^ = 0.82), indicating that they both played an important role in the SADF system.

### 3.5 SADF-related microbial community were widely distributed across global wastewater treatment plants

**Figure 6:**
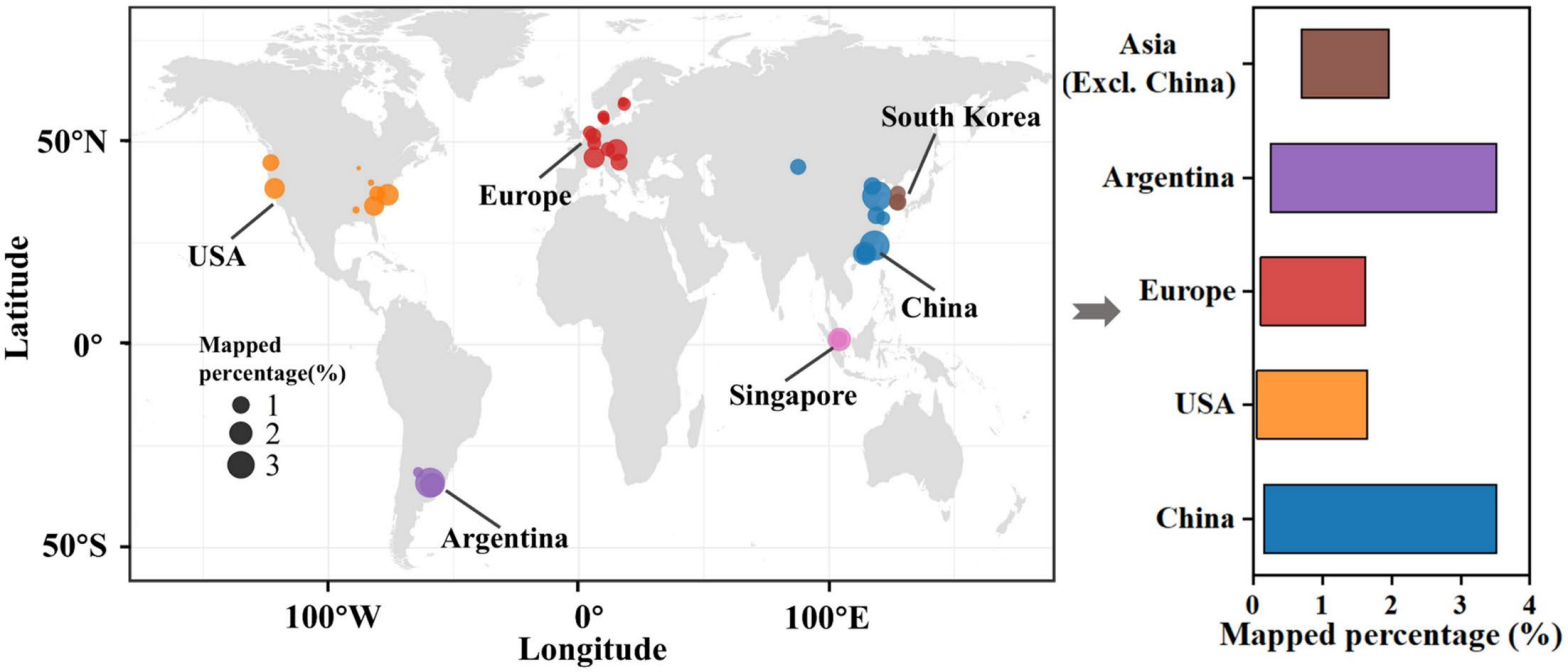
Global distribution of SADF-related bins identified in this study across wastewater treatment plants in 39 cities worldwide, calculated with mapped percentage.

DNA sequencing data were collected from 472 global activated sludge samples sourced from 57 WWTPs located in 39 cities across 12 countries and 4 continents (Figure 6). Raw reads were successively mapped with the assembled SADF-related genomic bins to assess whether the microbial community identified in this study displayed regional characteristics.

Notably, the average mapped percentage for 39 cities was 0.99%, with the highest value reaching 3.52% and the lowest being 0.06%. The highest value of 3.52% were from Zarate, Argentina, and Xiamen, China, respectively. These two locations are geographically distant, and the WWTP in Zarate is an industrial facility for petroleum refinery, while the one in Xiamen is a full-scale municipal WWTP. It is worth noting that a sample from Foshan, China, which is geographically close to Xiamen, had a mapped percentage of only 0.16%. These results strongly suggest that SADF-related microorganisms are widely distributed in global WWTPs.

This finding was further corroborated by statistical analysis. In this study, the 472 samples were classified into 5 groups according to their geographical locations: China, Asia (excluding China), Europe, USA, and Argentina. Intriguingly, one-way ANOVA analysis indicated no statistically significant differences between these groups (*p*-value > 0.05, see Supplementary Table 1). This implies that geographical location does not significantly affect the relative abundance of the SADF-related microbial community in WWTPs.

Previous studies have identified regional factors such as annual average temperature, humidity, and altitude as major determinants influencing the structure of activated sludge communities (Tian and Wang, 2020; Xia Y. et al., 2019; Xia Z. et al., 2019). However, this study revealed that for a specific subset of functional microorganisms in the activated sludge system, such as the SADF-related microbial community, their distribution remains relatively stable despite regional disparities. In other words, the widespread presence of the SADF-related microbial community in global WWTPs suggests its potential for indigenous enrichment and application of the SADF system on a global scale (Molari et al., 2023; Sriaporn et al., 2021). Previous studies have identified regional factors such as average annual temperature, humidity, and elevation as major determinants of microbial community composition in activated sludge systems (Tian and Wang, 2020; Xia Y. et al., 2019; Xia Z. et al., 2019). However, our findings highlight that certain functionally specialized microbial groups—such as those associated with SADF system—can exhibit relatively consistent global distribution patterns despite environmental and operational heterogeneity. This widespread presence underscores the potential for *in situ* enrichment and implementation of SADF-based processes across diverse geographic settings (Molari et al., 2023; Sriaporn et al., 2021). Nonetheless, it is important to acknowledge potential limitations that may affect the practical applicability of SADF systems across regions. Variations in wastewater composition, operational parameters (e.g., hydraulic retention time, oxidation–reduction potential), and indigenous microbial communities could influence system performance. Therefore, while the SADF-associated microbiota appears globally prevalent, their functional expression and ecological competitiveness may still be context-dependent, warranting further investigation under site-specific conditions.

## 4 Conclusion

This study conducted in-depth genomic and genetic analyses to explore the denitrification performance and the shift in the microbial community of the SADF system under different levels of sulfate salinity. The dominant genus, *Thiobacillus*, was found to increase in abundance as salinity rose. Co-occurrence network analysis indicated a larger number of positive correlations, which contribute to promoting stable denitrification performance. The upregulated stimulations in the nitrogen and sulfur metabolic pathways suggested that they exhibited strong adaptability patterns with the fast elevated salinity. Additionally, the SADF-related microorganisms were found to be widely distributed in global WWTPs, with no statistically significant differences detected among different groups. These findings could provide valuable insights for applications of the SADF system in treating wastewater with high sulfate salinity. Nevertheless, several limitations remain in the present study. First, the findings of our study should be interpreted with caution regarding their representativeness of long-term denitrification processes. Second, it should be noted that the study did not account for the potential influence of complex pollutants (e.g., heavy metals, organic compounds) typically present in real industrial wastewater. Third, the results primarily reflect taxonomic composition and inferred functional potential, rather than offering direct evidence of gene expression or metabolic activity. Future investigations should aim to address these limitations by extending the exposure duration to more accurately simulate long-term denitrification processes, conducting experiments using real industrial wastewater containing complex pollutants to enhance the applicability and reliability of the findings, and incorporating advanced analytical techniques (e.g., transcriptomics, proteomics, or metabolomics) to provide direct evidence of gene expression and metabolic dynamics, thereby offering a more comprehensive understanding of the underlying mechanisms.

## Data Availability

The original contributions presented in the study are publicly available. This data can be found here: https://www.ncbi.nlm.nih.gov/sra, accession number: PRJNA1265719.

## Footnotes

^1^ https://github.com/wwood/CoverM

^2^ https://www.r-project.org

^3^ https://www.ncbi.nlm.nih.gov/
