# Supplementary material for: Microbial response under sulfate stress in a sulfur-based autotrophic denitrification system
Source: Front Microbiol. 2025 Jun 4;16:1615317. doi: 10.3389/fmicb.2025.1615317 (PMC12174152; doi:10.3389/fmicb.2025.1615317)
Supplement: Supplementary file 1 [file Supplementary_file_1.docx]

1. **Supplementary Figures and Tables**

## Supplementary Tables

**Supplementary Table 1.** One-way ANOVA analysis result

| **ANOVA** | | | | | |
| --- | --- | --- | --- | --- | --- |
| Mapped percentage | | | | | |
|  | Sum of squares | df | Mean Square | F | Sig. |
| Between groups | 4.905 | 4 | 1.226 | 1.672 | 0.176 |
| Within groups | 28.597 | 39 | 0.733 |  |  |
| Total | 33.502 | 43 |  |  |  |

**Supplementary Table 2.** Enzyme codes and enzyme names

| **EC Number** | **Enzyme Name** |
| --- | --- |
| EC:[1.7.5.1](https://www.kegg.jp/entry/1.7.5.1)/1.7.99.- | nitrate reductase / nitrite oxidoreductase, alpha subunit |
| EC:[1.7.1.1](https://www.kegg.jp/entry/1.7.1.1) | nitrate reductase (NAD(P)H) |
| EC:[1.9.6.1](https://www.kegg.jp/entry/1.9.6.1) | nitrate reductase (cytochrome) |
| EC:[1.7.7.2](https://www.kegg.jp/entry/1.7.7.2) | ferredoxin-nitrate reductase |
| EC:[1.7.2.2](https://www.kegg.jp/entry/1.7.2.2) | nitrite reductase (cytochrome c-552) |
| EC:[1.7.1.15](https://www.kegg.jp/entry/1.7.1.15) | nitrite reductase (NADH) large subunit |
| EC:[1.7.7.1](https://www.kegg.jp/entry/1.7.7.1) | ferredoxin-nitrite reductase |
| EC:[1.7.2.6](https://www.kegg.jp/entry/1.7.2.6) | hydroxylamine dehydrogenase |
| EC:[1.7.2.1](https://www.kegg.jp/entry/1.7.2.1) | nitrite reductase (NO-forming) |
| EC:[1.7.2.8](https://www.kegg.jp/entry/1.7.2.8) | hydrazine dehydrogenase |
| EC:[1.7.2.7](https://www.kegg.jp/entry/1.7.2.7) | hydrazine synthase subunit |
| EC:[1.18.6.2](https://www.kegg.jp/entry/1.18.6.2) | vanadium-dependent nitrogenase alpha chain |
| EC:[1.18.6.1](https://www.kegg.jp/entry/1.18.6.1) | nitrogenase molybdenum-iron protein alpha chain |
| EC:[1.7.2.5](https://www.kegg.jp/entry/1.7.2.5) | nitric oxide reductase subunit B |
| EC:[1.7.2.4](https://www.kegg.jp/entry/1.7.2.4) | nitrous-oxide reductase |
| EC:[1.8.1.18](https://www.kegg.jp/entry/1.8.1.18) | CoA-dependent NAD(P)H sulfur oxidoreductase |
| EC:[1.13.11.55](https://www.kegg.jp/entry/1.13.11.55) | sulfur oxygenase/reductase |
| EC:[1.8.1.2](https://www.kegg.jp/entry/1.8.1.2) | sulfite reductase (NADPH) flavoprotein alpha-component |
| EC:[1.8.1.22](https://www.kegg.jp/entry/1.8.1.22) | dissimilatory sulfite reductase alpha subunit |
| EC:[1.8.5.6](https://www.kegg.jp/entry/1.8.5.6) | sulfite dehydrogenase (quinone) subunit SoeA |
| EC:[1.8.2.1](https://www.kegg.jp/entry/1.8.2.1) | sulfite dehydrogenase (cytochrome) subunit A |
| EC:[1.8.3.1](https://www.kegg.jp/entry/1.8.3.1) | sulfite oxidase |
| EC:[1.8.99.2](https://www.kegg.jp/entry/1.8.99.2) | adenylylsulfate reductase, subunit A |
| EC:[2.7.7.4](https://www.kegg.jp/entry/2.7.7.4) | 3'-phosphoadenosine 5'-phosphosulfate synthase |

## Supplementary Figures


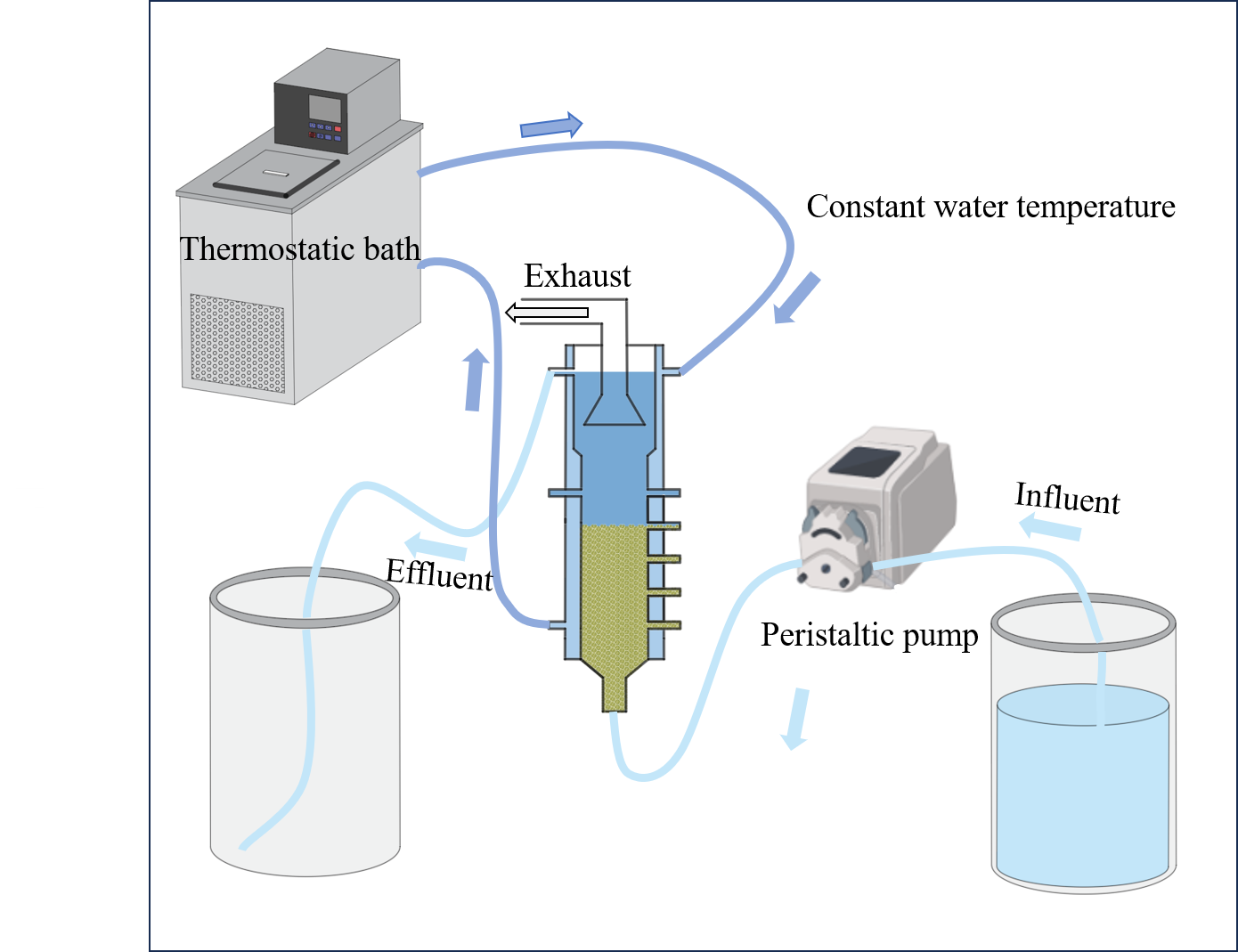


**Supplementary Figure 1.** A lab-scale upflow packed-bed SADF bioreactor.


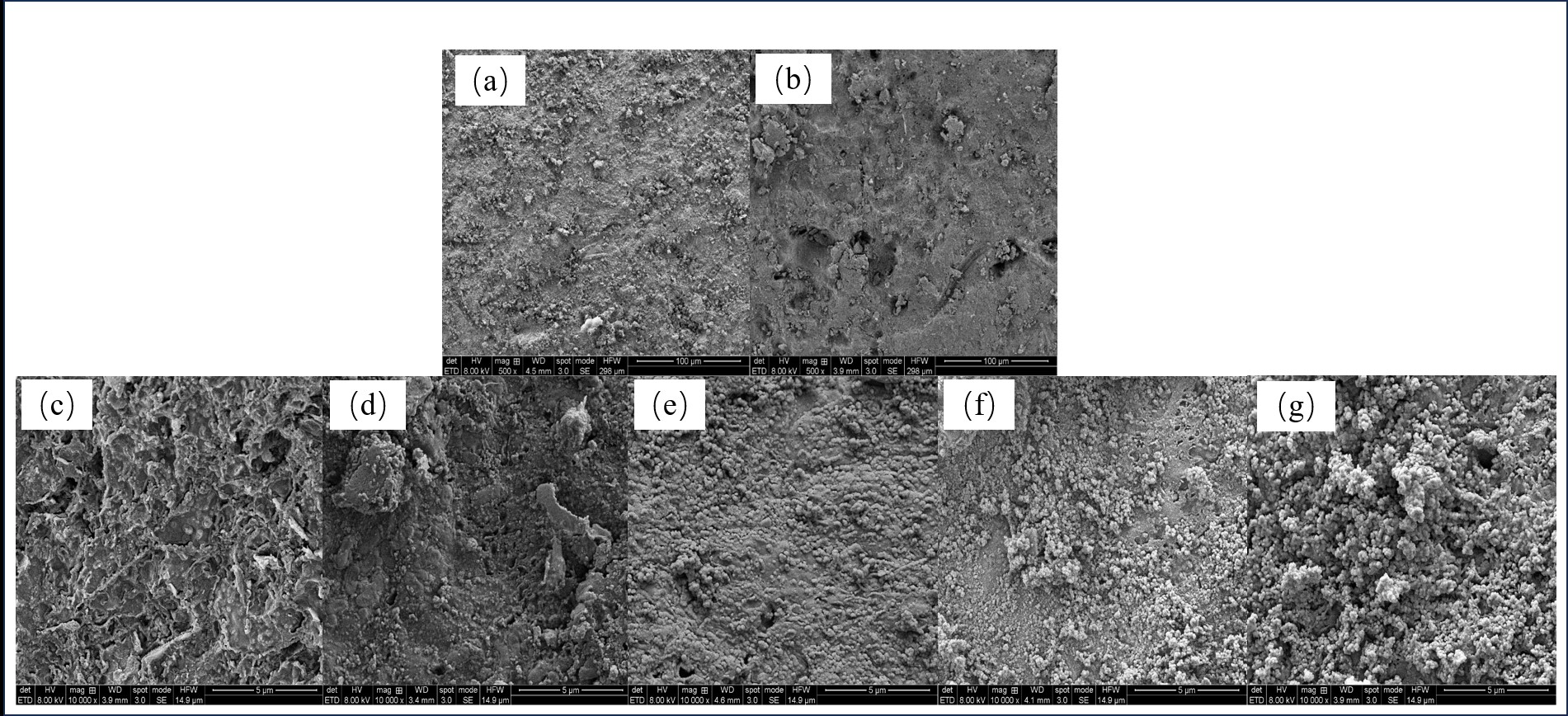


**Supplementary Figure 2.** Surface morphology of fillers in different salinity stages. (a) Unused filler (500x); (b) filler at salinity of 400 mg/L (500x); (c)-(g) filler at salinity of 400, 3000, 6000, 9000 and 12000 mg/L (10,000x), respectively.


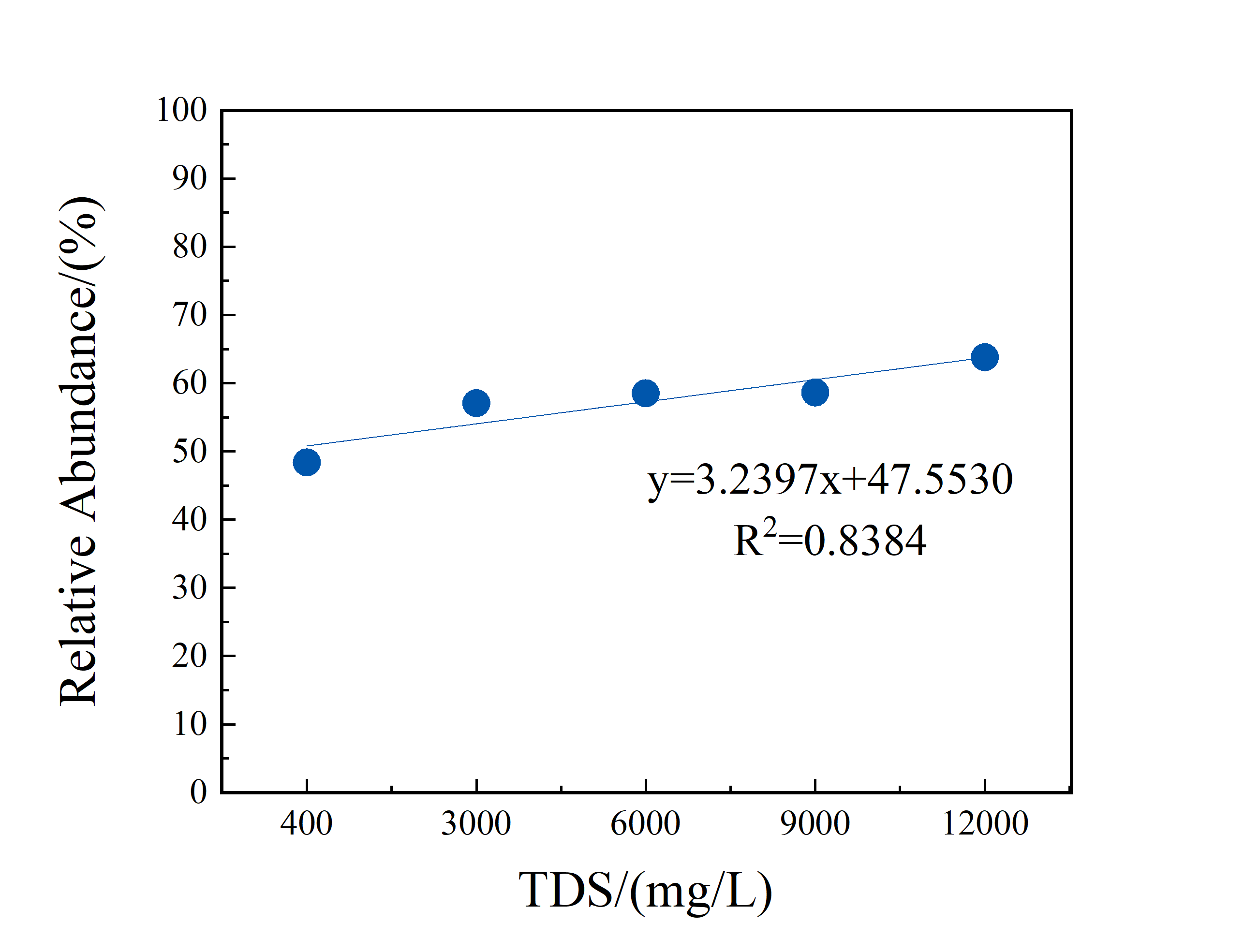


**Supplementary Figure 3.** *Thiobacillus* abundance fitting plot.


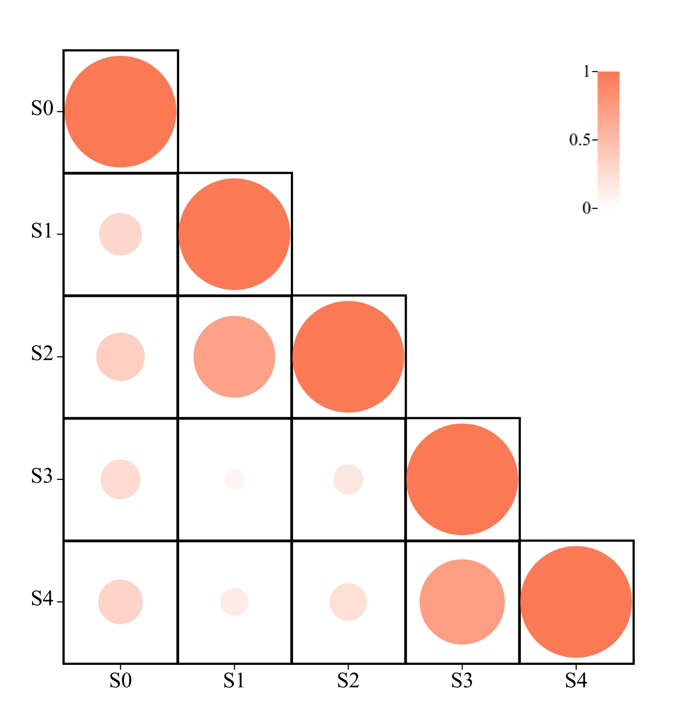


**Supplementary Figure 4.** Heat-map diagram of inter-sample correlation analysis (Color shade and circle area represent the magnitude of Spearman correlation coefficients, where brighter color and larger circles indicates larger positive correlation coefficients between each pair).
